# Altered Gut Microbiota in Children With Hyperuricemia

**DOI:** 10.3389/fendo.2022.848715

**Published:** 2022-04-27

**Authors:** Xin Yuan, Ruimin Chen, Ying Zhang, Xiangquan Lin, Xiaohong Yang

**Affiliations:** Department of Endocrinology, Genetics and Metabolism, Fuzhou Children’s Hospital of Fujian Medical University, Fuzhou, China

**Keywords:** hyperuricemia, children, gut microbiota, *Alistipes*, *Bilophila*

## Abstract

**Background:**

In adults, gut dysbiosis may contribute to the pathogenesis of gout. However, the characteristics of gut microbiota in children with hyperuricemia (HUA) in the absence of clinical gout have not been explored.

**Objective:**

This present study analyzed the gut microbiota in children with HUA as compared to controls (Con) and explored bacterial associations that may account for differences.

**Methods:**

A total of 80 children were enrolled in this study; they were divided into HUA and Con according to the level of serum uric acid (UA). The composition of gut microbiota was investigated by 16S rRNA high-throughput sequencing.

**Results:**

Principal coordinate analysis revealed that gut microbiota of the HUA group was clustered together and separated partly from the Con group. There was no difference in alpha-diversity between the two groups. However, Spearman’s correlation analysis revealed that serum UA level positively correlated with genera *Actinomyces, Morganella*, and *Streptococcus*, and negatively associated with the producers of short-chain fatty acids (SCFAs), such as *Alistipes, Faecalibacterium*, and *Oscillospira*, and the sulfidogenic bacteria *Bilophila.* The members of the genera *Alistipes* and *Bilophila* in the Con group were significantly more prevalent than the HUA subjects. Compared to the Con cohort, metabolic pathway predictions found that the superpathways of purine nucleotide *de novo* biosynthesis were decreased in HUA subjects, whereas the superpathway of purine deoxyribonucleoside de gradation was increased.

**Conclusion:**

The composition of the gut microbiota in children with HUA differs from Con. Although causality cannot be established, modification in the microbiota that produces SCFA and sulfide may promote HUA.

## Introduction

The prevalence of childhood and adolescent obesity is a global plight with wide-ranging health consequences. Many obese children already harbor one or more metabolic disturbances such as hyperuricemia (HUA), dyslipidemia, and type 2 diabetes (1, 2). Of these, HUA is recognized as a certain risk factor for hypertension and diabetic kidney disease in adults with type 2 diabetes and also likely in the general population (3, 4). Indeed, an elevated baseline serum uric acid (UA) foretells the development of hypertension and elevated urinary albumin excretion independently (5). As observed in several disorders, especially obesity, the gut microbiome is distinct from healthy controls (6). However, the metabolic relationship between the gut microbiota and asymptomatic HUA is unexplored in children.

Healthy humans excrete UA *via* two principal ways: about 25% through the GI tract, and 75% renal. As a consequence of the former pathway, exposure to UA by intestinal microbiota may alter the microbiome composition. Furthermore, the gut microbiota, such as *Escherichia coli*, can participate in purine and UA metabolism (7). Compositional bacterial alterations could plausibly contribute to the advancement of UA (8–11) and thereby be a potential therapeutic target (12). Several studies regarding the gut microbiota have been reported in adults with gout; however, only one study in clinically healthy HUA adult male patients involved the structural and functional alterations of gut microbiota (13). Exploring the seminal pathogenesis of HUA in the microbiome of children may provide insights into its targeted treatment (14). This study explored the fecal microbial signature of children with HUA as compared to healthy controls.

## Patients and Methods

### Study Population

The Ethics Committee of the Fuzhou Children’s Hospital of Fujian Medical University approved this study, and patient participation required written informed consent.

This study was limited to residents of Fujian province; recruitment period was from September 2017 to March 2018. A total of 80 patients between 5 and 15 years of age who were admitted to Fuzhou Children’s Hospital of Fujian Medical University for monitoring of growth and development were recruited. The clinical diagnosis and prior blood results were obtained from hospital records. The exclusion criteria were as follows: children with any endocrine disease associated with obesity, chronic disease of cardiovascular, respiratory, kidney, and other systems, recent antibiotic therapy, any acute gastrointestinal illness 1 month prior to the enrollment, or hospitalization (>24 h) at any point half a year prior to enrollment. Smoking history was also exclusionary.

A brief medical history was obtained by questionnaire. Standardized survey including anthropometric and demographic data were completed by all participants.

### Anthropometric and Biochemical Assessment

Height and weight were measured with calibrated scale and height gauge by trained nurses. BMI-Z scores were calculated using Chinese reference values (15). Puberty status was defined according to the Tanner scale by pediatric endocrinologists. All participants were required to maintain their usual dietary habits at least 3 days, and then fasted for 12 h before blood sampling. For non-pubertal children, the fasting blood samples were collected for routine standard of care. For pubertal children, the blood samples were collected for biochemical and sex hormone levels (testosterone for boys and estradiol for girls). Blood samples were stored at −80°C and analyzed within 2 weeks. Biochemical assessment including fasting serum UA, glucose, cholesterol, triglycerides, blood urea nitrogen (BUN), and serum creatinine (Cr) was determined using standard laboratory methods (Beckman Coulter AU5800, USA) with specific reagents. Serum testosterone and estradiol levels were measured by chemiluminescent immunoassays (IMMULITE 2000, Siemens Healthcare Diagnostics Products Limited, Germany) with specific reagents.

### Definition of HUA

HUA is defined as UA > 6 mg/dl (16).

### Fecal Sample Collection and Processing

Fecal samples of participants were collected within 3 days after blood collection in standard stool collection tubes, and then stored at −80°C within 2 h until assayed.

### 16S rRNA Gene Tag Sequencing and Analysis

We used MagPure Stool DNA KF kit B (Magen, China) to extract DNA from thawed fecal samples according to the manufacturer’s protocols. The extracted products underwent 1% agarose gel electrophoresis. A Qubit Fluorometer and Qubit^®^ dsDNA BR Assay kit (Invitrogen, USA) were used for DNA yield quantification. Extracted DNA samples were stored at −20°C prior to Illumina Miseq sequencing analysis.

The V3–V4 region of the 16S rRNA genes was amplified by PCR as the previous study described (17). The Agilent 2100 bioanalyzer (Agilent, USA) was used to qualify the libraries. The validated libraries were sequenced on an Illumina MiSeq platform (BGI, Shenzhen, China) by generating 2 × 300-bp paired-end reads following Illumina’s standard pipeline.

Quality trimmed reads were demultiplexed and paired-end reads were joined both using Quantitative Insights into Microbial Ecology 2 software (QIIME2, version 2019.10) with default settings (18). Demultiplexed sequences were subjected to quality filtering and denoising in DADA2 *via* q2-dada2 and obtain the amplicon sequence variant (ASV) feature sequences (19). ASVs were aligned with mafft, and a rooted phylogenetic tree was generated with fasttree2 (*via* q2-phylogeny) (20). The 16S rDNA sequences were screened for chimeras by mapping to gold database (v20110519). ASVs were taxonomically classified using Qiime2-feature-Classifier trained on the database Greengene (v 13.8) (21).

### Statistical Analysis

The Statistical Package for the Social Sciences software version 23.0 (SPSS Inc. Chicago, IL, USA) was used to analyze the clinical data. Kolmogorov–Smirnov analysis tests were used to test the normality of the data. The independent samples *t*-test was assessed for comparisons between groups depending on the data distribution. Pearson’s chi-squared test compared the ratios. *p*-values < 0.05 were considered statistically significant. Wilcoxon rank-sum test (“wilcox.test” package in R) was utilized to detect differentially enriched microbes (genus and species-level) between HUA and Con groups (FDR < 0.05). Non-parametric permutational multivariate analysis of variance (PERMANOVA) was conducted using the “vegan” package to assess the driving factors of microbiota composition (FDR < 0.05). For bacterial biomarker identification, discriminant analysis (DA) based on univariate ANOVAs, Fisher’s coefficient, and leave-one-out classification were performed to define a model based on the capability of OTUs to discriminate the two groups of study participants. A cross-validation (CV) test was applied to verify the capability of the entire OTUs set to discriminate the two groups. We adopted the Wilks’ Lambda test with *p* < 0.05 filter to select OTUs and tested their discriminatory power in correctly classifying groups by applying the linear operating characteristic curve (ROC curve) to evaluate the microbial markers. An area under the ROC curve (AUC) greater than 0.7 was deemed statistically significant to discriminate the two groups.

Alpha- and beta-diversity was accessed by software QIIME2 (v2019.7) to measure the diversity within and between microbial communities, respectively (18). Linear discriminant analysis and cladograms identified differentially abundant taxa between groups using the LDA effect size (LefSe) on Galaxy platform (22). PICRUSt2 with default parameters was applied to predict metagenome and functional composition from 16S rRNA gene surveys (23) and to annotate gene expression pathways according to Kyoto Encyclopedia of Genes and Genomes (KEGG). Possible enriched bacterial function was established between groups by a two-sided Welch’s *t*-test and multiple test correction Benjamini-Hochberg FDR using Statistical Analysis of Metagenomic Profiles (STAMP) (*q*-value filter > 0.05) (24).

## Results

### Study Participants

The mean age of the 80 children was 9.73 ± 1.91 years (ranging from 5.7 to 14.1 years), and 48 were boys. The levels of UA in boys and girls were 387.98 ± 95.96 and 349.38 ± 69.39 μmol/L, respectively, and there was no statistical difference between genders (*p* = 0.054). There were 21 pubertal boys and 19 pubertal girls. Most of the pubertal subjects were in early or middle stage of puberty. The median of testosterone level in pubertal boys was 53.5 pg/dl (*n* = 15), and the median of estrogen level in pubertal girls was 20.32 pg/ml (*n* = 14). Forty children were identified as HUA and 40 served as control (Con). Age, BMI, and level of serum UA in the HUA group were significantly higher than Con (all *p* < 0.05). Importantly, however, there was no difference in BMI-Z between the cohorts. The levels of serum BUN and Cr of all the participants were normal. The levels of high-density lipoprotein cholesterol were significantly lower in the HUA group (1.40 ± 0.31 vs. 1.56 ± 0.33 mmol/L, *p* = 0.033), yet there was no statistical difference in fasting plasma glucose, triglycerides, total cholesterol, and low-density lipoprotein cholesterol (all *p* > 0.05, **Table 1**).

**Table 1:** Anthropometric profiles and laboratory measurements.

	HUA (*n* = 40)	Con (*n* = 40)	*t *(chi-squared) value	*p*-value
Age (years)	10.34 ± 1.86	9.12 ± 1.77	2.992	0.004
Male (%)	67.5	52.5	(1.875)	0.119
BMI (kg/m^2^)	25.13 ± 4.15	22.25 ± 5.01	2.793	0.007
BMI-Z	2.41 ± 1.04	2.00 ± 1.58	1.363	0.178
UA (μmol/L)	489.62 ± 58.87	330.86 ± 51.03	11.755	<0.001
FPG	4.93 ± 0.55	5.01 ± 0.46	−0.672	0.504
TG	1.24 ± 0.85	0.95 ± 0.40	1.934	0.057
TC	4.40 ± 0.72	4.51 ± 0.87	−0.624	0.535
LDL-c	2.45 ± 0.52	2.48 ± 0.67	−0.262	0.794
HDL-c	1.40 ± 0.31	1.56 ± 0.33	0.796	0.033

HUA, hyperuricemia; Con, control; BMI, body mass index; BMI-Z, BMI standard deviation Z score; UA, uric acid; FPG, fasting plasma glucose; TG, triglycerides; TC, total cholesterol; LDL-c, low-density lipoprotein cholesterol; HDL-c, high-density lipoprotein cholesterol.

### Microbiota Profiles

**Figure 1:**
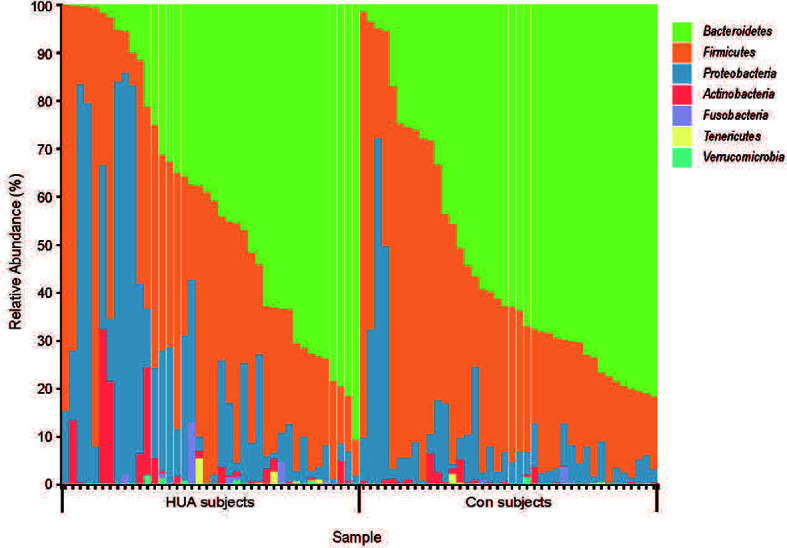
The taxa-bar of gut microbiota in HUA and Con subjects at the phylum level. HUA, hyperuricemia; Con, control.

Overall, we obtained a total of 659,252 reads (average of 8,240 counts per sample) from the 80 fecal samples by 16s rRNA gene sequencing. Overall, 134 ASVs were identified, among which 121 ASVs have more than 2 counts. Taxonomic assignment of the ASV revealed a total of 7 phyla across all samples (**Figure 1**).

#### Correlations Between Serum UA Level and Bacterial Abundance

Spearman’s correlation analysis revealed that serum UA level positively correlated with genera *Actinomyces, Morganella*, and *Streptococcus*, and negatively associated with genera *Alistipes, Bilophila, Faecalibacterium, Oscillospira, Parabacteroides*, and *Phascolarctobacterium* (all *p* < 0.05, **Table 2**).

**Table 2:** Spearman’s correlation table on OTUs and serum uric acid level.

	*R*	Correlation label	*p*-value
*Actinomyces*	0.363	pos	0.001
*Alistipes*	0.353	neg	0.001
*Bilophila*	0.358	neg	0.001
*Faecalibacterium*	0.227	neg	0.042
*Morganella*	0.239	pos	0.033
*Oscillospira*	0.282	neg	0.011
*Parabacteroides*	0.257	neg	0.022
*Phascolarctobacterium*	0.244	neg	0.029
*Streptococcus*	0.256	pos	0.022

pos, positive; neg, negative.

#### Abundance Profiling in HUA and Con Subjects

**Figure 2:**
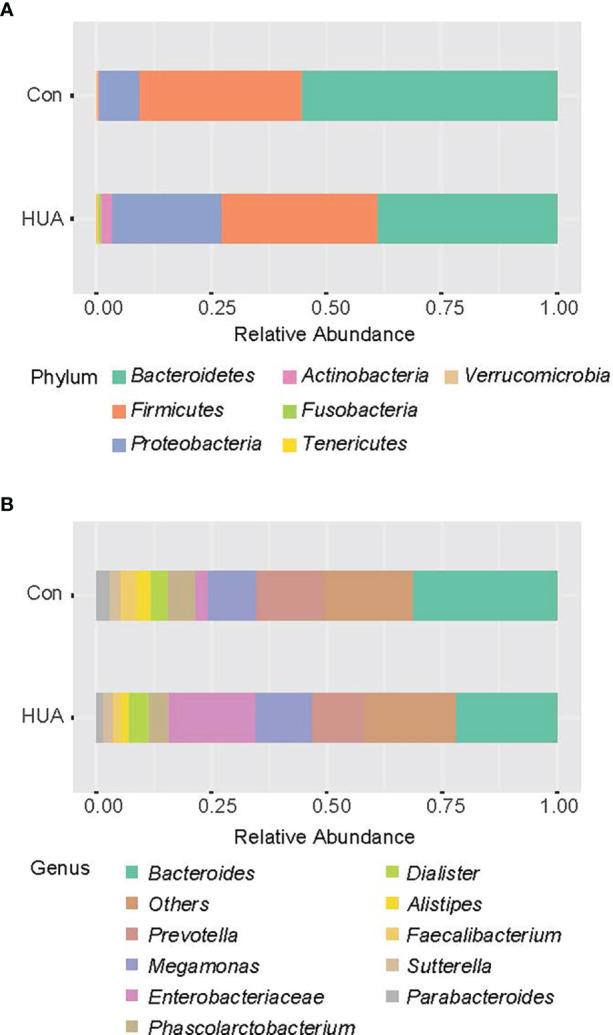
Bar chart representing Mann–Whitney *U*-test results on OTUs grouped in phyla **(A)** and in genus **(B)** of the HUA and Con subjects (showing top 10 taxa). HUA, hyperuricemia; Con, control.

The Mann–Whitney *U*-test was applied to analyze the relative abundances of phyla between groups. Compared to the Con subjects, an increase of *Proteobacteria* and a reduction of *Bacteroidetes* in the HUA subjects was observed (both *p* < 0.05, **Figure 2A** and **Table 3**).

**Table 3:** The mean relative abundance of gut microbiota in HUA and Con subjects with significant differences.

Level	Bacteria	HUA	Con	*Z*	*p*-value
Phylum	*Bacteroidetes*	0.390	0.554	−2.146	**0.032**
	*Proteobacteria*	0.237	0.085	−2.444	**0.015**
Genus	*Actinomyces*	0.0004	0.0000	−3.137	**0.002**
	*Streptococcus*	0.0185	0.0036	−2.575	**0.010**
	*Alistipes*	0.0188	0.0323	−2.392	**0.017**
	*Bilophila*	0.0027	0.0062	−3.418	**0.001**

Bold was used when P < 0.05.

At the genera level (counts less than 10 were merging), Mann–Whitney *U*-test revealed that genera *Actinomyces* and *Streptococcus* were more prevalent in HUA subjects than Con, whereas genera *Alistipes* and *Bilophila* were more prevalent in the Con subjects (all *p* < 0.05; **Figure 2B** and **Table 2**).

#### Alpha- and Beta-Diversity in HUA and Con Subjects

**Figure 3:**
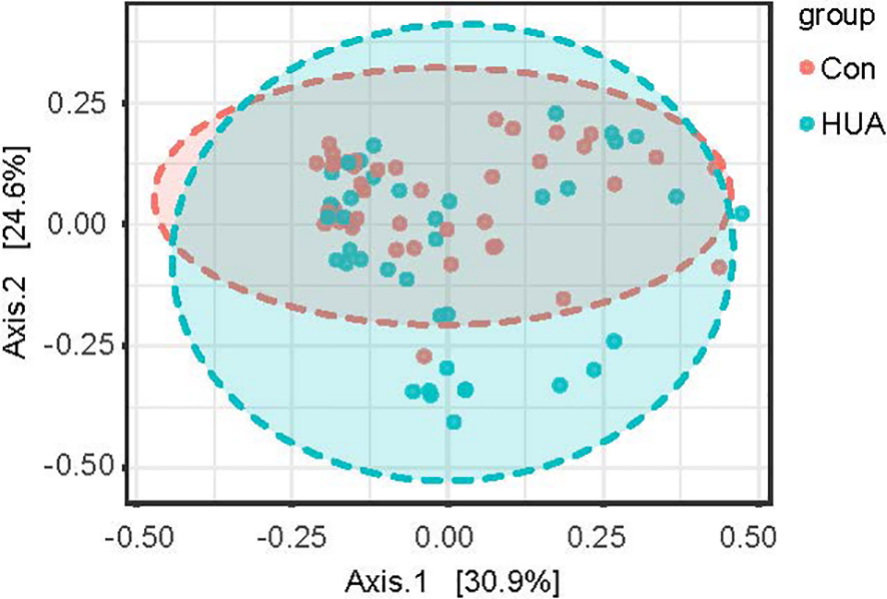
PCoA plot of HUA and Con subjects. HUA, hyperuricemia; Con, control.

Regarding alpha-diversity, the Shannon diversity index, observed OTUs, Faith’s phylogenetic diversity, and Pielou’s evenness based on ASV distribution were analyzed between groups. No difference was observed between the groups (all *p* > 0.05, **Table S1**). Regarding beta-diversity, Bray–Curtis distance, Jaccard distance, Unweighted-unifrac, and Weighted-unifrac were analyzed, and principal coordinate analysis (PCoA) based on statistical method PERMANOVA and Bray–Curtis metric distances of the genus composition revealed separation of the two groups, and the level of UA explained 25.5% of the variation in the microbiota (*F*-value: 2.8611; *R*^2^: 0.035383; *p-*value < 0.008, **Figure 3** and **Table S2**).

#### Bacterial Taxa Differences in HUA and Con Subjects

**Figure 4:**
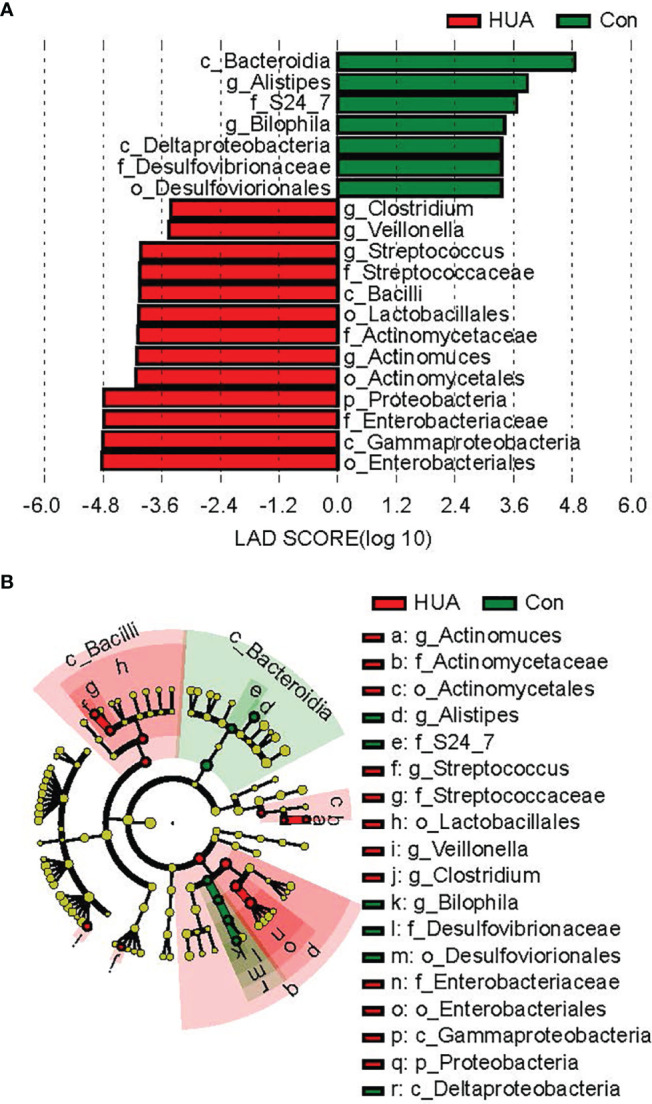
Differential biomarkers in HUA and Con subjects by LefSe analysis (α value = 0.05, logarithmic LDA score threshold = 2.0). HUA, hyperuricemia; Con, control. In the LEFse tree, different colors indicate different groups. Note colored in a group color shows an important microbe biomarker in the group and the biomark name will list in the upper right corner. The yellow notes represent the biomarker which do not show any importance in groups. **(A)** LDA plot of the differential bacterial by LEfSe analysis; **(B)** clodagram of the differential bacteria lby LEfSe analysis.

By LEfSe analysis, the phylum *Proteobacteria*, genus *Clostridium, Actinomyces, Streptococcus*, and *Veillonella* in HUA subjects were significantly more prevalent compared to Con subjects. In contrast, genus *Alistipes* and *Bilophila* in the Con subjects were significantly more prevalent than the HUA subjects (all *p* < 0.05, **Figure 4**).

### Microbial Biomarkers

Based on univariate ANOVAs, Fisher’s coefficient, and leave-one-out classification, DAs were performed to define a model to discriminate the HUA and Con subjects by bacterial abundance. At the phylum level, the relative abundance of *Proteobacteria* could correctly classify 68.8% of the original grouped subjects by DA and 66.3% of cases by cross-validation test (**Table S3**). At the genus level, genus *Bilophila* and *Faecalibacterium* could correctly classify 66.3% of the original grouped subjects by DA and 65% of cases by the CV test (**Table S4**).

**Figure 5:**
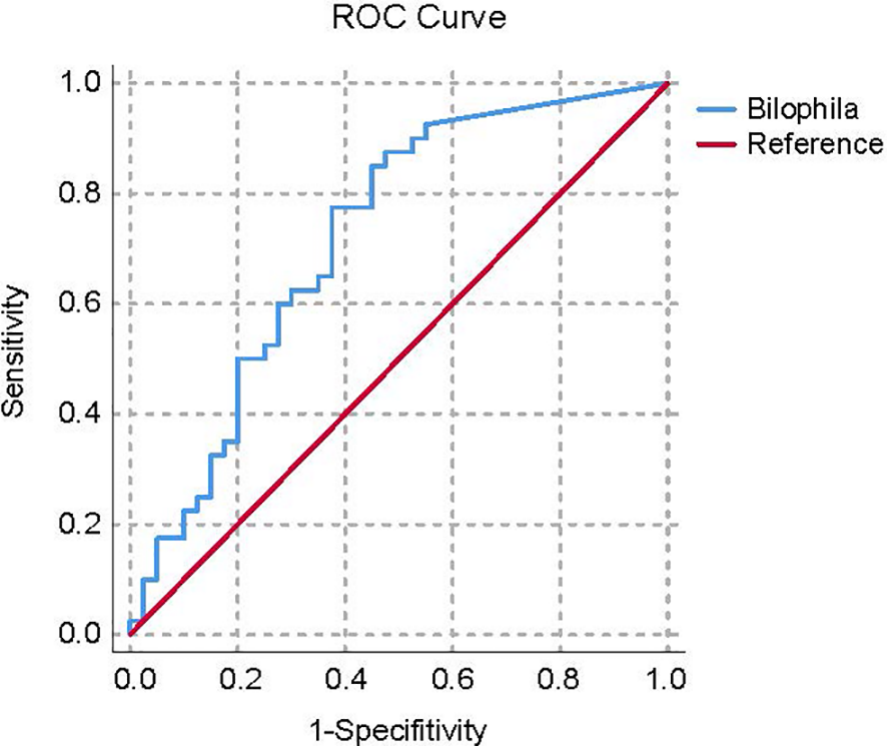
ROC curve plots of the bacteria able to discriminate Con subjects from HUA subjects. HUA, hyperuricemia; Con, control.

By applying the average AUC, the discriminatory power of the ASV at the genus level in correctly classifying the two groups was further tested. The AUC of genus *Bilophila* for the Con subjects from the general population was 0.720 (**Figure 5**).

### Metabolic Pathway Predictions

**Figure 6:**
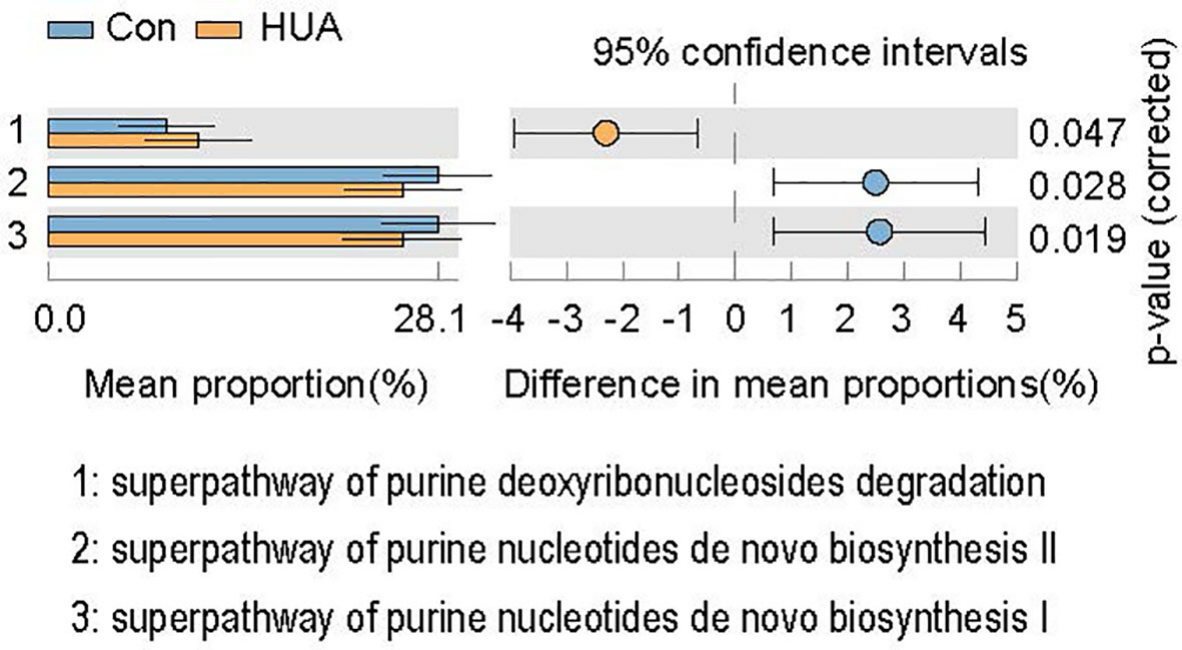
KEGGs biomarkers of HUA and Con subjects. HUA, hyperuricemia; Con, control.

In total, PICRUSt2 analysis corrected by multiple test correction Benjamini-Hochberg FDR revealed 79 KEGG pathways based on the composition of the gut microbiota (**Figure 4** and **Table S4**). Notably, the purine metabolism pathways, including superpathway of purine nucleotides *de novo* biosynthesis I and superpathway of purine nucleotides *de novo* biosynthesis II, were attenuated in HUA subjects, and the superpathway of purine deoxyribonucleosides degradation was increased compared to the Con subjects (*p* < 0.05, **Figure 6**).

## Discussion

Pertinent metabolic interactions between gout and the gut microbiota exist such that certain microbiota can regulate the synthesis and catabolism of purine and UA (25). Some bacteria produce short-chain fatty acids (SCFAs) (26, 27) whereas others modify the number and distribution of UA transporters (28). Each of these studies found a robust correlation between HUA and the gut microbiota. In our two metabolically distinct cohorts (each *n* = 40), we found taxonomic variations and shifts in the HUA group regarding bacterial urate metabolism. In HUA children, *Actinomyces* and *Streptococcus* are enriched, whereas *Alistipes* and *Bilophila* are depleted. This is the first study attempt to assess the characteristics of gut microbiota in children with asymptomatic HUA.

In a prior report, the taxonomic gut microbiota of adult gout patients had richness indices including Chao1, observed species and ACE that were significantly decreased compared to controls, while Shannon and Simpson diversity indices and beta-diversity were similar between the study groups (29). Conversely, another study (13) reported significant alpha- and beta-diversity in the HUA group compared with controls. In our study, despite no significant difference in alpha-diversity of the gut microbiota in those with HUA, the beta-diversity was significantly reduced. Different disease status (gout and HUA), weight, medication exposure, diet, and age may account for disparities in the findings in these studies.

Besides the diversity of the gut microbiota in our HUA subjects, the community of the gut microbiota also differed between the two groups, a finding concordant to that of Sheng S (13). Patients with gout have abundant *Bacteroides* in their gut microbiomes compared to healthy individuals (9–11). *Bacteroides* possess an enrichment of the enzyme 5-hydroxysourate hydrolase, pivotal in gut uricolysis, and hence could regulate serum urate concentrations (30). On the other hand, elevated *Bacteroides* abundance may be a consequence of gout disease per se (31). Regardless, there are scant studies of asymptomatic individuals with HUA and their gut flora, which could provide prescient insight into the pathogenesis of the HUA.

Both the activity of thew immune system and the anti-inflammatory effects could be mediated by SCFAs in chronic diseases (32, 33). Moreover, the gut microbiota can affect the excretion of UA by the production of SCFAs (26–28). Consistent with previous reports, we found that the serum UA level was negatively associated with the abundance of genus *Alistipes, Faecalibacterium, Oscillospira, Parabacteroides*, and *Phascolarctobacterium* (34, 35). These bacteria are deemed as salutary microbiota insofar as they produce SCFAs (36–38). Using an experimental murine model of gout, and introducing a high-fat diet known to induce elevated levels of SCFAs, an accelerated resolution of the inflammatory response was observed, inferring a role for SCFAs in HUA-related inflammation (26). The proliferation of those salutary bacterial members may be reduced by HUA, which possibly contributes to the development of gout (9).

Regarding butyrate-producing bacteria *Alistipes* and sulfidogenic bacteria *Bilophila*, both can affect the host immune system in an antithetical fashion (39). The hypothesis that loss of gut butyrate producers may be altered in gout was buttressed by computational predictions (31). Furthermore, elevated H_2_S production consequent to sulfur-rich L-cysteine feeding from *Faecalibacterium* may play a role in promoting disease. Microbiome results may confirm a community interplay in which synthesis of the potentially inflammatory metabolite H_2_S (40–42) may be supported by health-promoting taxa such as *Faecalibacterium* that cross-feed metabolites to a disease-promoting taxa (32). These predictions suggested that sulfur-containing amino acids may be a contributor, or a marker, in the perturbed microbiota of patients with HUA.

In this study, we found that the genus *Clostridium, Veillonella*, and *Streptococcus* were more prevalent in HUA subjects than Con, consistent with a prior study in which *Clostridium* and *Veillonella* positively correlated with serum UA (31). The genus *Clostridium* is considered as an obese-associated genus in both Chinese (43) and Danish (44) populations. Zeng et al. (34) reported that the superabundance of genera *Clostridium* was shared by obese patients with various metabolic disorders. However, this association was not observed in our previous study analyzing the gut microbiota in obese children with diverse metabolic status (glucose, lipid, and blood pressure). Intriguingly, gout can affect the compositional stability of intestinal *Clostridium*, and alters *Clostridium* species and quantity (45). We observed that the genera *Clostridium* was more prevalent in HUA subjects, and most of the study population enrolled in our study were obese (BMI-Z was 2.20 ± 1.34). Following which, we opine that the disparate metabolic abnormalities in patients with obesity are important confounding factors when analyzing the gut microbiota in children with HUA.

Jones et al. (46) assessed dietary macronutrients using 24-h diet recalls in fifty-two obese individuals, reporting that the genus *Streptococcus* was inversely associated with dietary fructose intake. Granting that, the intake of fructose is just one confounding factor that could affect the concentration of serum UA (47). As a southeast coastal region, many other relevant factors such as excessive consumption of meat, seafood, and sugar-sweetened soft drinks (48), and even the single-nucleotide polymorphisms (49) in urate exchange gene, are possibly disparate in our geographic region. Although the microbes belonging to the genus *Streptococcus* have been implicated in the development of various metabolic disorders (50), the link between fructose intake and the entire genus *Streptococcus* has not been explored.

*Proteobacteria* is known as a potential diagnostic signature of dysbiosis and risk of disease (51). In this study, we found that the abundance of *Proteobacteria* increased significantly in the HUA group, which was consistent with a prior study that reported that the phylum *Proteobacteria* was more abundant in patients with tophaceous gout than in controls (29). This change might play a crucial role in the development of gout (52). Furthermore, the abundance of *Bacteroidetes* decreased significantly in the HUA group in this study. Recently, Kim et al. (53) analyzed the differences in microbiota composition between patients with gout and those with asymptomatic HUA, and found that the asymptomatic HUA group exhibited a significantly low proportion of *Bacteroidetes* compared to the gout group. However, another study reported that after PCR amplification, the number of bands of *Bacteroides* in patients with primary gout was not reduced compared with normal individuals (45). Since there is no study comparing the characteristics of gut microbiota in asymptomatic HUA, gout, and healthy individuals at the same time, it is still impossible to analyze the role of *Bacteroidetes* in different stages of the disease.

Previous studies suggested that *Parabacteroides* may afford an unexplained protective effect on liver metabolism (54). Interestingly, we found that this bacterium negatively associated with the serum level of UA. This finding is novel but could be due to age, diet, or other factors.

It has been reported that the levels of UA were similar between genders before puberty, but UA levels are higher in boys after puberty than in girls due to the action of sex hormones. It is generally believed that testosterone can inhibit the excretion of UA while estrogen can promote the excretion of UA (55). In our study, there was no statistical difference between genders. However, the sample size of pubertal subjects was limited; as a result, the effect of gender on UA levels cannot be established.

There are limitations in this study. First, being cross-sectional by design, the causality between the gut microbiome and HUA could not be verified. Furthermore, given that our subjects reside in the same region, a multicenter study from dissimilar regions with diverse diets and environments would be enlightening.

## Conclusion

Our study details the structural and functional alterations of gut microbiota in obese children with HUA. Correlation between UA and gut microbiome implicated the SCFA-producing *Alistipes* and the sulfidogenic bacteria *Bilophila* as contributors to circulating UA. Based on our preliminary findings, the potential role of the gut microbiota, especially those that generate SCFAs or sulfides, on HUA is inferential. Nonetheless, our correlations between gut bacterial species and purine-related pathways with HUA hold promise of specific microbiota-targeted therapies in the prevention or treatment of gout.

## Data Availability Statement

The original contributions presented in the study are publicly available. These data can be found here: NCBI, PRJNA787036.

## Ethics Statement

The studies involving human participants were reviewed and approved by the Ethics Committee of the Fuzhou Children’s Hospital of Fujian Medical University. Written informed consent to participate in this study was provided by the participants’ legal guardian/next of kin.

## Author Contributions 

XY drafted the initial manuscript. RC conceptualized and designed the study, and reviewed and revised the manuscript. YZ and XHY collected cases. XL did the laboratory testing. All authors contributed to the article and approved the submitted version.

## Funding

This study was supported by the Technology Innovation Team Train Project of Fuzhou Health Committee in China (2016-S-wp1), and sponsored by key Clinical Specialty Discipline Construction Program of Fuzhou, Fujian, China (201610191) and Fuzhou Children’s Medical Center (2018080310).

## Conflict of Interest

The authors declare that the research was conducted in the absence of any commercial or financial relationships that could be construed as a potential conflict of interest.

## Publisher’s Note

All claims expressed in this article are solely those of the authors and do not necessarily represent those of their affiliated organizations, or those of the publisher, the editors and the reviewers. Any product that may be evaluated in this article, or claim that may be made by its manufacturer, is not guaranteed or endorsed by the publisher.
