# Supplementary material for: Altered Gut Microbiota in Children With Hyperuricemia
Source: Front Endocrinol (Lausanne). 2022 Apr 27;13:848715. doi: 10.3389/fendo.2022.848715 (PMC9091909; doi:10.3389/fendo.2022.848715)
Supplement: Supplementary file 1 [file Table_1.docx]

**Table S1** Comparison of alpha-diversity between HUA and Con subjects

| alpha-diversity index | H | P value |
| --- | --- | --- |
| Shannon | 0.653 | 0.419 |
| Observed OTUs | 1.414 | 0.232 |
| Faith’s phylogenetic diversity | 3.203 | 0.073 |
| Pielou’s evenness | 0.441 | 0.507 |

**Table S2** Comparison of beta-diversity between HUA and Con subjects

| beta-diversity index | pseudo-F | P value |
| --- | --- | --- |
| Bray-Curtis distance | 1.879 | 0.003 |
| Jaccard distance | 1.208 | 0.011 |
| Unweighted-unifrac | 2.340 | 0.008 |
| Weighted-unifrac | 2.798 | 0.050 |

**Table S3.** Discriminant analysis table based on statistically different OTUs at phyla level in HUA and Con subjects

| **predicted group membership** | | | | | |
| --- | --- | --- | --- | --- | --- |
|  |  | group | HUA | Con | total |
| Original | count | HUA | 21 | 19 | 40 |
|  |  | Con | 6 | 34 | 40 |
|  | % | HUA | 52.5 | 47.5 | 100.0 |
|  |  | Con | 15.0 | 85.0 | 100.0 |
| Cross-validated | count | HUA | 19 | 21 | 40 |
|  |  | Con | 6 | 34 | 40 |
|  | % | HUA | 47.5 | 52.5 | 100.0 |
|  |  | Con | 15.0 | 85.0 | 100.0 |

**Table S4.** Discriminant analysis table based on statistically different OTUs at genus level in HUA and Con subjects

| **predicted group membership** | | | | | |
| --- | --- | --- | --- | --- | --- |
|  |  | group | HUA | Con | total |
| Original | count | HUA | 28 | 12 | 40 |
|  |  | Con | 15 | 25 | 40 |
|  | % | HUA | 70 | 30 | 100 |
|  |  | Con | 37.5 | 62.5 | 100 |
| Cross-validated | count | HUA | 28 | 12 | 40 |
|  |  | Con | 16 | 24 | 40 |
|  | % | HUA | 70 | 30 | 100 |
|  |  | Con | 40 | 60 | 100 |

**Table S5.** KEGGs biomarkers in HUA and Con subjects.

| pathway | Con: mean rel. freq. (%) | Con: std. dev. (%) | HUA: mean rel. freq. (%) | HUA: std. dev. (%) | p-values  (corrected) |
| --- | --- | --- | --- | --- | --- |
| superpathway of purine deoxyribonucleosides degradation | 8.524 | 3.447 | 10.819 | 3.810 | 0.047 |
| superpathway of purine nucleotides de novo biosynthesis I | 28.111 | 4.077 | 25.542 | 4.279 | 0.019 |
| superpathway of purine nucleotides de novo biosynthesis II | 28.114 | 3.870 | 25.623 | 4.206 | 0.028 |
